# Non-invasive methods to assess seed quality based on ultra-weak photon emission and delayed luminescence

**DOI:** 10.1038/s41598-024-74207-9

**Published:** 2024-11-05

**Authors:** Adriano Griffo, Stefanie Sehmisch, Frédéric Laager, Andrea Pagano, Alma Balestrazzi, Anca Macovei, Andreas Börner

**Affiliations:** 1 Department of Biology and Biotechnology ‘L. Spallanzani’, University of Pavia, Via Ferrata 9, Pavia, PV 27100 Italy; 2 Leibniz Institute of Plant Genetics and Crop Plant Research, Corrensstr. 3, Gatersleben, 06466 Seeland, Saxony-Anhalt Germany; 3 SUPER Lab, 26160 Bad Zwischenahn, Germany

**Keywords:** Delayed luminescence, Leguminosae, Machine learning, Non-invasive assessment, Seed quality, Ultra-weak photon emission, Plant sciences, Optics and photonics

## Abstract

Seed quality is the set of physical, genetic, and physiological characteristics, reflecting the overall germination potential. Maintaining an optimal seed quality is essential for agriculture and seed banks to preserve genetic diversity. Compared to conventional methods (e.g., germination tests), non-invasive approaches allow a more sustainable and rapid evaluation of seed quality but this is limited by high costs. The measurement of ultra-weak photon emission (UPE) and delayed fluorescence (DL), defined as biological phenomena potentially related to the physiological status of living systems, may represent a suitable approach to estimate seed quality. To test this hypothesis, seeds of five agriculturally relevant legume species (*Phaseolus vulgaris* L., *Lathyrus sativus* L., *Cicer arietinum* L., *Pisum sativum* L., and *Vicia faba* L.), stored at different conditions (room temperature or -18 °C) for several years, were analysed using a LIANA© prototype to collect data regarding DL and UPE occurring after UV excitation. The obtained data were integrated with germination parameters which underline species-specific behaviours in response to storage conditions. The prediction models show variable efficiency in classifying seeds based on germination which underline species-dependent links between photon emission and seed quality. Therefore, these measurements represent novel, non-invasive, and rapid approaches to evaluate seed quality.

**Supplementary Information:**

The online version contains supplementary material available at 10.1038/s41598-024-74207-9.

## Introduction

 Seed quality is the set of genetic, physiological, and physical features of seeds (https://www.seedtest.org/*).* Since seed quality reflects the overall germination potential and influences crop production, its evaluation is crucial for seed companies and consumers to both optimize economic profits and increase the final crop yield^1^. Crop production must be increased to meet the ZERO HUNGER target relative to the Sustainable Developmental Goal SDG#2 of the 2030 UN Agenda for Sustainable Development (https://www.un.org/sustainabledevelopment/). In a scenario where crop production must be sustainably enhanced, novel methods to assess seed quality can substantially increase the availability of high-quality seeds, with a positive effect on agriculture costs and food production.

Monitoring seed quality is very important for many stakeholders, including germplasm banks, breeders, agronomists, seed companies and consumers^1^. The use of high-quality seeds is a proxy of the seed market, which translates into a continuous increase in the commercial seed market trends (Seeds Global Market Report 2024)^2^. Several methods are available for seed quality testing. Conventional germination, electrical conductivity, seedling growth, triphenyltetrazolium chloride (TTC) test, and accelerated ageing are approved by the International Seed Testing Association (ISTA) and constitute the most used approaches so far^3–5^. However, these methods have some considerable limitations, including invasiveness, extensive amount of test work required, long test periods, low accuracy and operators biases^1^. To efficiently measure seed quality and avoid the waste of resources, novel methods to assess it and subsequent quality attributes are necessary and highly sought. To this purpose, non-invasive optical techniques, including machine vision^6,7^, NIR (Near InfraRed), Raman spectroscopies^8,9^, thermal, X-ray, and hyperspectral imaging^10–12^, have been developed and applied to test seed quality. Despite their advantages in gaining high-throughput information in a rapid, non-invasive, and accurate manner, the high cost and the complexity of these technologies limit their large-scale use^13^. So far, no universal approach has been developed to assess seed quality in a rapid, accurate, economic, and non-destructive manner. Therefore, the search for such methods is still highly required and requested.

Ultra-weak photon emission (UPE) is defined as the luminescence generated from the production of electronically excited species produced from the oxidative processes^14^. Since oxidative reactions are solely responsible for the spontaneous generation of photons, this phenomenon potentially occurs in living cells of all organisms, from bacteria to animals^15^. Although the origin and the nature of the electronically excited species are partially unknown and very complex, Cilento and Adam^16^ described the concept of electronic excitation and the electronic configuration of molecules on the ground state T_0_, the singlet state S_1_, and the triplet state T_1_, which stand at the basis of this process. The transition of electrons that occurs in common oxidation and reduction reactions results in the transition of the molecule into different energy states (T_0_, S_1_, T_1_) due to the different energy of the electrons exchanged. Photon release marks the transition of a molecule from an excited state (S_1_ or T_1_) to the starting state T_0_^15,16^. Most of the pathways that generate electronically excited species involve radical species as well as oxygen molecules for the electronic transition^16^, confirming the importance of ROS (reactive oxygen species) in this process. UPE can be spontaneous, where the release of photons during the oxidative processes happens without any external stressors or stimuli, or it can be induced by stress and oxidative factors that promote oxidative reactions^15,17^. Another phenomenon related to UPE is Delayed Luminescence (DL), defined as the long-term afterglow of biological systems after illumination^18^. The DL general trend is characterized by an initial peak of intensity (in terms of the number of photons released per time) followed by a rapid decay. DL can occur for seconds or milliseconds, depending on the time of the inductor and the used system^19^.

In recent years, the possible link between UPE, DL, and the physiological state of biological systems has gained more interest from the scientific community. Because UPE and DL are generated from oxidative processes occurring during metabolic reactions, it is connected with ROS production^20–23^, molecules involved in many biological processes^24^, including seed quality and germination^25^. UPE and DL have been successfully applied to evaluate food quality^26^. Similarly, these phenomena have been linked to germination, pointing to a connection between the physiological state and photon release^27,28^. In the context of seed evaluation, DL has been applied to detect additional features like water content^29^ and viability^30^. Although, these initial reports provide insights into the UPE and DL application in seed biology, the complex features of the phenomena and their implications, require further investigation. Therefore, the aim of this study was to assess the use of UPE and DL as tools to predict seed quality taking into consideration multiple legume species and accessions. Germination performance was evaluated in five legume species (*Phaseolus vulgaris* L., *Lathyrus sativus* L., *Cicer arietinum* L., *Pisum sativum* L., and *Vicia faba* L.), using seeds stored at different conditions (room temperature or -18 °C) for more than ten years. The study has focused on legumes as these are economically important crops characterized by high nutrient content and have important agronomic applications given their symbiosis with nitrogen-fixing bacteria. The species with the highest number of accessions available at the IPK genebank collection were selected to take into account intraspecific variability. UPE and DL occurring after UV excitation were collected using a LIANA© prototype and the generated data were integrated with the germination parameters using machine learning algorithms to generate prediction models to estimate seed quality in a non-invasive manner.

## Results

### Development of an experimental system for UPE and DL data collection

**Fig. 1:**
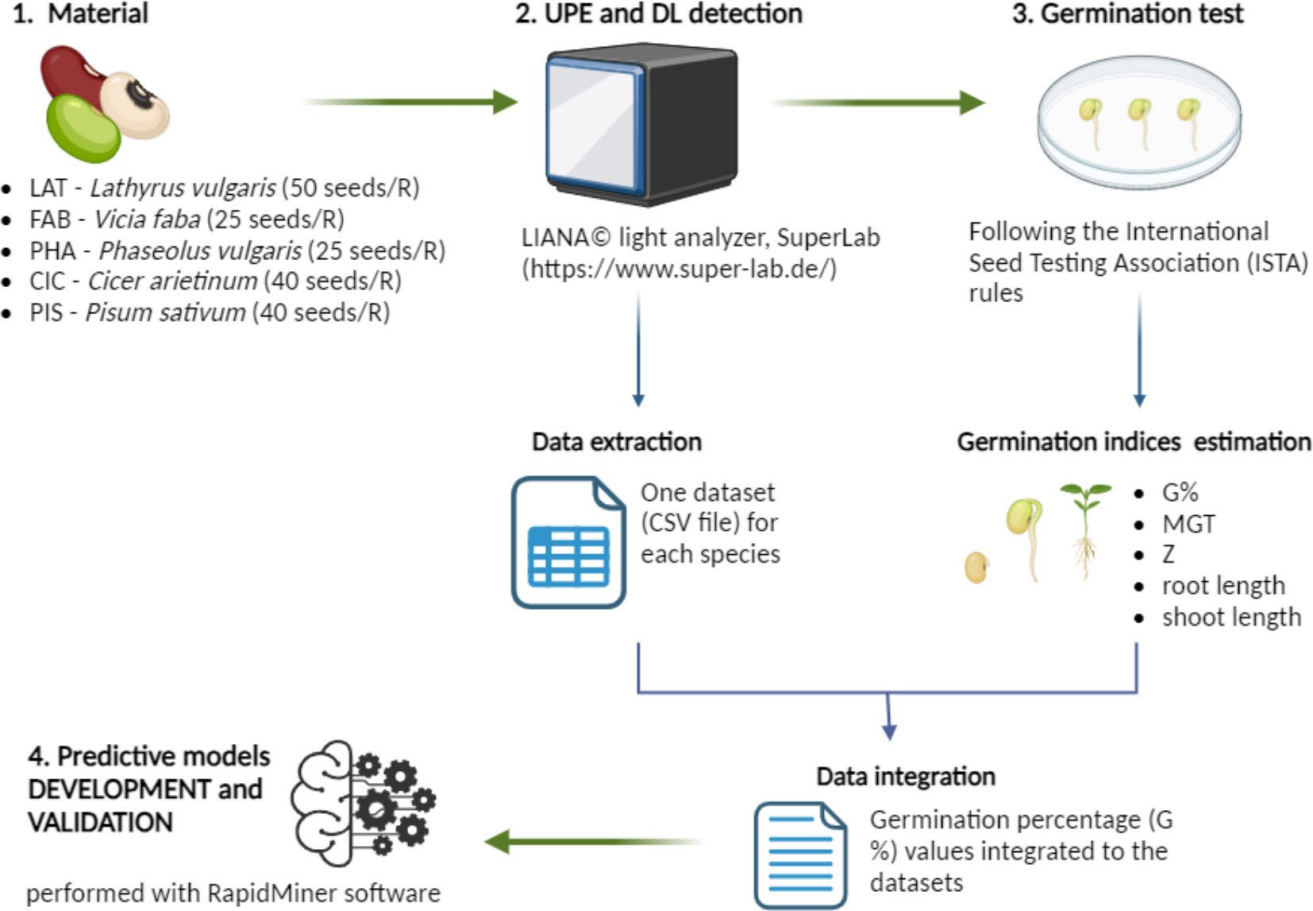
Schematic representation of the experimental system. Multiple accessions of seeds of five legume species stored at room temperature (Ambient) or -18 °C (Cold) for more than ten years were ordered in different biological replicates (R) based on their size. The replicates were analyzed with the LIANA© light analyzer and subsequently used for germination test, following the ISTA rules for each species. At the end of the germination test, several indices (G%, Z, MGT, root and shoot length) were calculated and the germination percentage data was integrated into the extracted CSV file containing the UPE and DL data for the same replicates. The complete datasets were independently used for the development of machine learning models for germination prediction using RapidMiner^®^ software.

The experimental system proposed in this study is based on using seeds stored at different conditions: a seed bank optimal storage conditions at -18^o^C (hereby defined as Cold) and an ambient room temperature (22–24 ^o^C) storage (hereby defined as Ambient). The investigated legume species (*P. vulgaris*,* L. sativus*,* C. arietinum*,* P. sativum*,* V. faba*) and multiple accessions were stored for more than ten years under Ambient and Cold conditions prior to use. Information describing the species and accessions, including time of harvest, years of storage, and origin, are provided in Supplementary Tables S1-S5. Figure 1 depicts the experimental model along with the analyses carried out to obtain the final dataset for the predictive models developed through machine learning approach. Seed samples were used to detect UPE and DL and subsequently germinated to assess a set of indices indicative of seed quality. To develop the predictive models, seed samples were classified into two groups (optimal, non-optimal) based on the germination percentage, where optimal germination ranges between 80 and 100% while below 80% is considered as non-optimal.

## Germination performance under different storage conditions

**Fig. 2:**
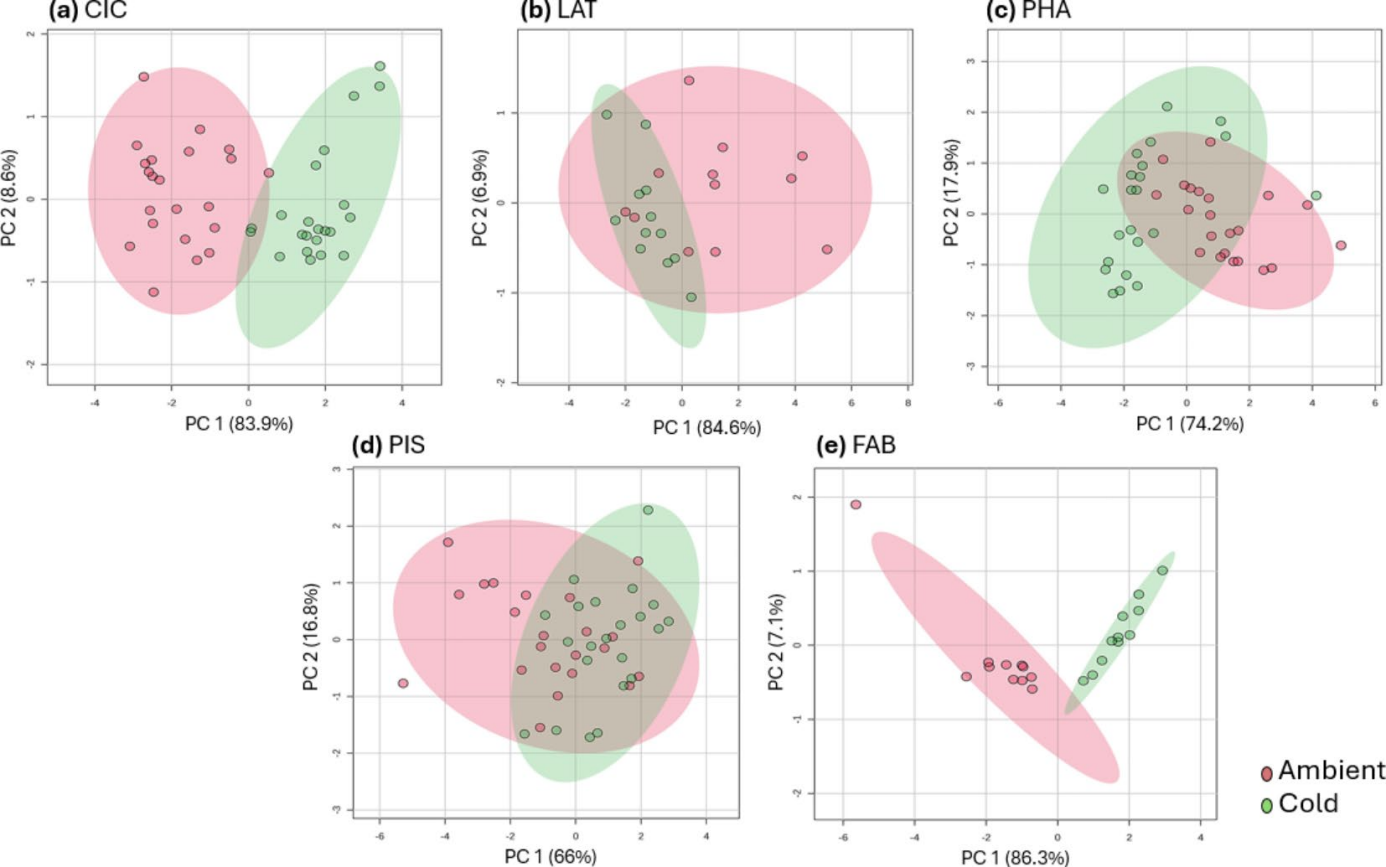
PCA score plots generated using the germination data, and LIANA© parameters gathered from seeds stored at Ambient (red) and Cold (greed) storage conditions. (**a**) *Cicer arietinum* (CIC). (**b**) *Lathyrus sativus* (LAT). (**c**) *Phaseolus vulgaris* (PHA). (**d**) *Pisum sativum* (PIS). (**e**) *Vicia faba* (FAB).

Germination tests were performed to estimate the effect of storage conditions on germination performances on each of the investigates species and selected accession. Given the high amount of data, the values (mean ± st.dev.) of germination percentage (G%), mean germination time (MGT), synchronicity index (Z), root and shoot length are provided for each species/accession in the Supplementary dataset. For the overall representation of these data, a principal component analysis (PCA) was performed (Fig. 2). The data depicts two scenarios; first, represented by *C. arietinum* (CIC) and *V. faba* (FAB), where there is a distinct clustering between the Ambient and Cold groups, and second, represented by *P. vulgaris* (PHA), *L. sativus* (LAT), and *P. sativum* (PIS) where the two clusters are overlapping. For CIC and FAB, the majority of accessions stored under cold conditions present an optimal G% (80–100%) while storage at room temperature resulted in reduced G% below 80% (e.g., 53–7% in CIC648 and CIC702; 76–0% in FAB129 and FAB6975, see Supplementary dataset). For the second scenario, multiple seed samples stored under Ambient conditions show optimal values of G%, therefore the distinction between Ambient and Cold is less pronounced. Among the PIS accession, 38 samples present germination above 80% while 6 samples are classified in the group below 80%. For the PHA accessions, 33 have optimal germination while the remaining 11 present non-optimal germination. Lastly, LAT is divided into 13 accessions with optimal germination and 11 accessions with non-optimal performance (Supplementary dataset). The remaining germination indices follow a similar pattern as G% in the different species, accessions, and storage conditions, thus supporting the divergent PCA clustering.

**Fig. 3:**
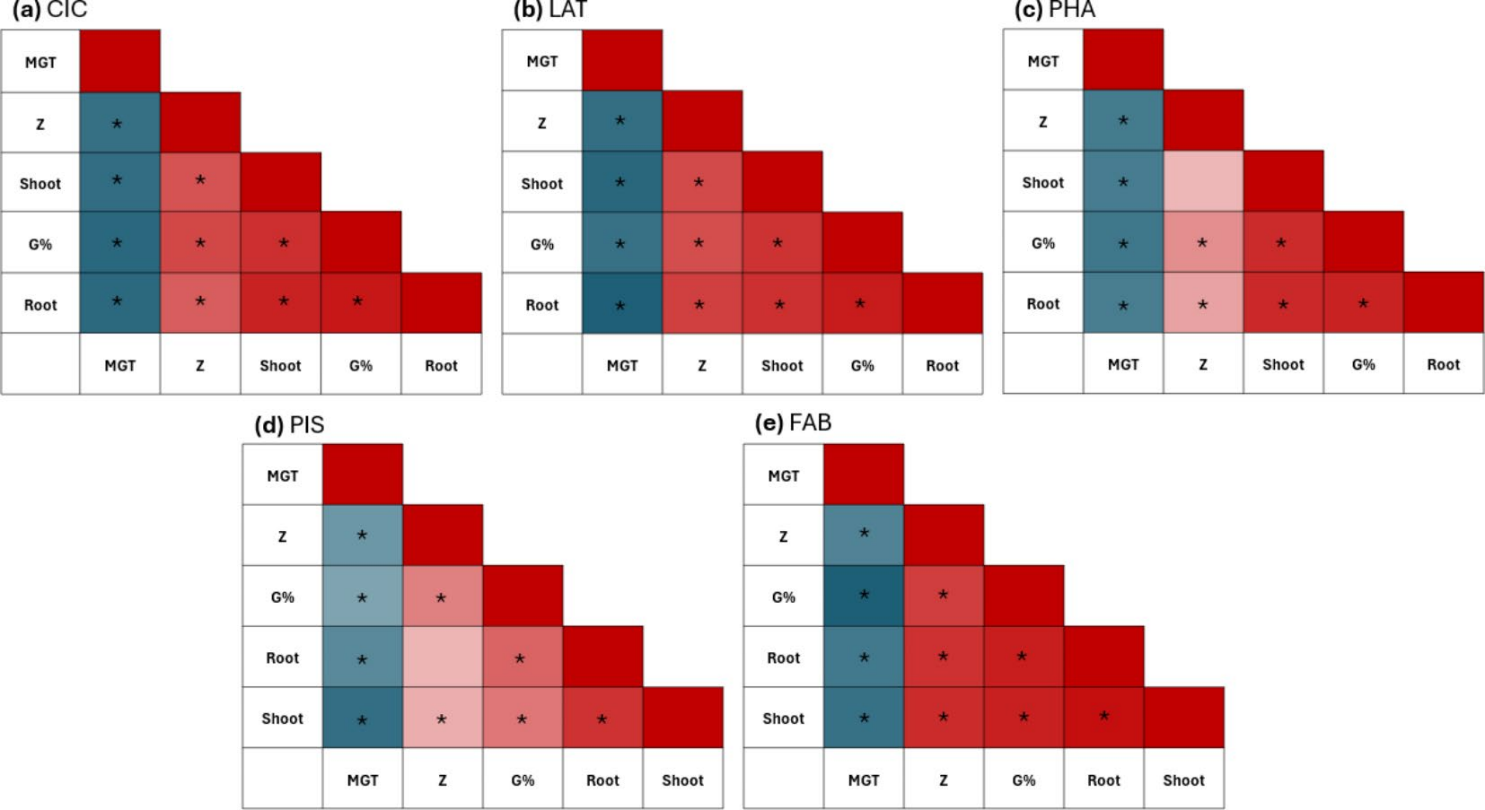
Pearson correlation analysis based on using (G%), mean germination time (MGT), synchronicity index (Z), root and shoot length. (**a**) *Cicer arietinum* (CIC). (**b**) *Lathyrus sativus* (LAT). (**c**) *Phaseolus vulgaris* (PHA). (**d**) *Pisum sativum* (PIS). (**e**) *Vicia faba* (FAB). Statistically significant correlations are indicates with an asterisk (*p ≤ 0.05).

To evaluate the degree of correlation between the different germination indices, the Pearson coefficient *r* was calculated and graphically represented in Fig. 3. Similar trends of correlations are observed among all the investigated species. MGT is negatively correlated with all the other parameters which are positively correlated to each other. This suggests that seeds with optimal germination percentage are also characterized by high speed and synchrony, in addition to enhanced seedling growth.

This first step of the study allowed to characterize a system with different germinative performances that can be used to test novel methods dedicated to predict seed viability in a non-invasive manner.

## Analysis of photon counts in correlation with germination percentage

**Fig. 4:**
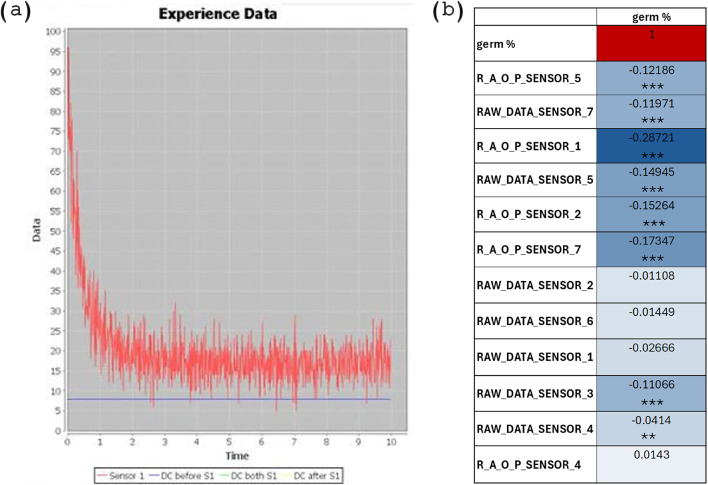
Photon count determination. (**a**) Example of a time course of phonon emission curve as provided by the integrated software of LIANA© prototype. (**b**) Pearson correlation table of LIANA© parameters indicating the photon counted (raw and corrected) through each sensor (1–7) with the percentage of germination (G%), using the data of all the tested species. Statistically significant correlations are indicated by asterisks (*p < 0.05; **p < 0.01; ***p < 0.001). R_A_O_P, real amount of photons, obtained by subtracting the photons counted before and after the measurement time and by multiplying by correction factors, which consider the position of the PMTs, the filter used, and the overlapping of wavelengths with other PMTs; RAW_DATA, initial photon counts not corrected.

The LIANA© prototype, used in this study for UPE and DL detection, collects 1334 parameters reflecting the entire photon release phenomena. The prototype includes seven sensors (photomultiplier tubes, PMTs) that allow the detection of photons at different wavelengths (see Methods paragraph UPE and DL detection). An example of a time course of phonon emission curve is shown in Fig. 4a. The “RAW_DATA” parameter corresponds to the total of photon counted for each sensor while “R_A_O_P” corresponds to the corrected value of photons, obtained by subtracting the photon courted during dark count and by multiplying to correction factors. The values of “RAW_DATA” and “R_A_O_P” are provided in the Supplementary dataset for each sensor per species. To investigate if the values of photon counts (in terms of “RAW_DATA” and “R_A_O_P” for each sensor) can be related to G%, a correlation analysis was performed (Fig. 4b). Significant negative correlations can be observed for the following parameters: RAW_DATA sensors 3, 4, 5, 7, and R_A_O_P sensors 1, 2, 5, 7. Among these, data from “R_A_O_P sensor 1”, showing the most relevant correlation value (dark blue in Fig. 4b), was used to plot photon counts for each accession in relation to germination (Fig. S1). To generate these plots taking account of the Amb and Cold groups, fold-change (FC, Amb/Cold) values were used for both parameters. Among species and accessions, the ratio of photon counts is heterogeneous; however, a higher number of accessions show FC values below 1, reflecting a lower emission of photons from Amb groups. Despite this trend, the FC values regarding G% do not indicate a precise trend between these parameters. Overall, this indicates that considering only the photon counts does not explain the complete UPE and DL phenomena and their possible link with germination performance. Given the high number of parameters provided by the prototype, it is therefore highly required to use more complex data analysis systems, such as machine learning.

## Application of predictive models for seed classification

**Fig. 5:**
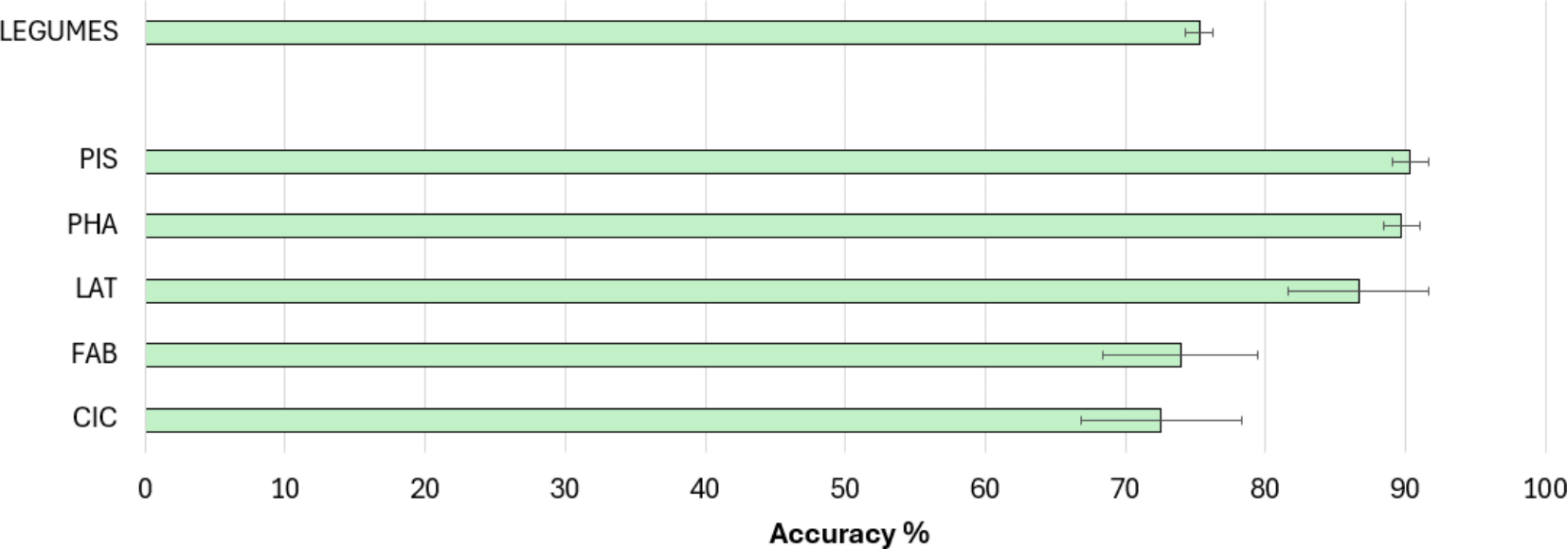
Percentage (%) of accuracy for the prediction models developed using the RapidMiner software based on germination data. Validation was performed using the “cross-validation” operator (number of folds = 10). CIC, *C. arietinum*; LAT, *L. sativus*; PHA, *P. vulgaris*; FAB, *V. faba*; PIS, *P. sativum*.

**Fig. 6:**
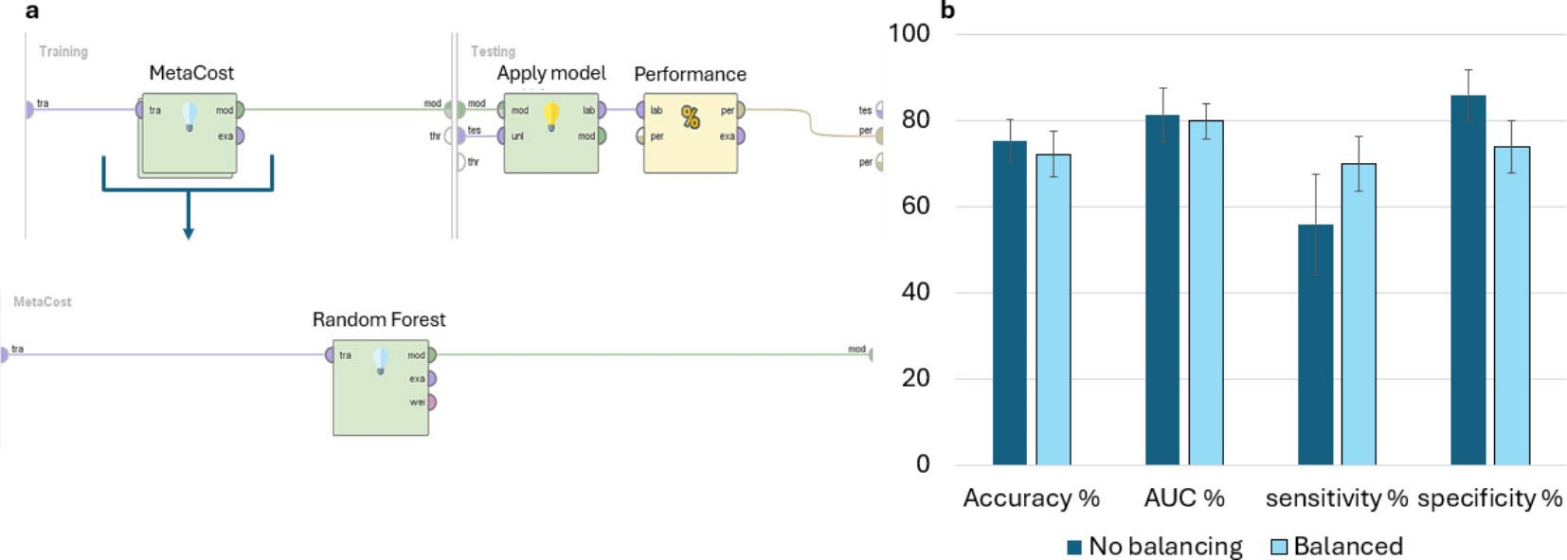
MetaCost operator utilization on CIC learning model. (**a**) Structure of the cross-validation operator, including the MetaCost operator into the Training subprocess and the Random Forest classifier, included into the MetaCost operator. (**b**) Histograms showing the classification parameter percentages (Accuracy, AUC, sensitivity, and specificity) calculated on the CIC predictive model with (Balanced, light blue) and without (No Balancing, dark blue) MetaCost operator.

Using the UPE/DL data provided by the prototype and the germination performance classification, a predictive model was formulated. To train the models, the samples (records) were classified into two quality classes ranging from optimal (80–100%) and non-optimal (< 80%) germination. The 80% threshold was selected based on previous studies on genebank accessions dedicated to understand how long seeds can retain their viability over extended periods of uncontrolled temperature or non-optimal conditions^31^. Figure 5 shows the accuracies of the prediction models. The models developed for the single species datasets (CIC, LAT, PHA, PIS, FAB) indicate different accuracy values. Models developed using *V. faba* and *C. arietinum* datasets presented a moderate accuracy (73.96% and 72.5%, respectively), while the accuracy of the other species reached higher values (above 85%). Subsequently, to uniformize these data, the dataset “Legumes” was obtained by unifying the collections of data from the single species with the operator “append” of RapidMiner software. This operator merges two or more datasets with the same attributes building a new combined set. The accuracy of the prediction model developed with the “Legumes” dataset is around 75.29% (Fig. 5), indicating a good prediction efficiency. Table 1 presents other classification metrics that describe the overall efficiency of predictive models. While positive predictive value (PPT) and negative predictive values (NPT) follow the trend exhibited by the accuracy parameter, sensitivity and specificity percentages reflect a critical issue in classifying seed samples in the appropriate group in most models. In particular, the model developed from the PIS dataset registers the lowest value of specificity (approximately 15.83%). One potential solution is represented by the MetaCost operator of RapidMiner, which makes the prediction cost-sensitive by utilizing a specified cost matrix (configured by the operator)^32,33^. In the learning model depicted in Fig. 6a, the MetaCost operator was used as trial to improve the sensitivity value of the predictive model developed from the CIC dataset (55.91%, Table 1), configuring a cost matrix that assigns a cost of 5 and 4 to false negatives and false positives, respectively. The results are presented in Fig. 6b, which shows the main classification parameters of the model with (Balanced) and without (No Balancing) MetaCost operator. The histogram illustrates a notable improvement of the sensitivity, which was the objective of MetaCost operator utilization. However, this improvement is balanced by a reduction in specificity, while the accuracy and the AUC values exhibited a slight impact from the optimization process. In general, the application of machine learning allowed a more efficient and a complete handling of the parameters provided by the LIANA© prototype to estimate seed quality.

**Table 1:**
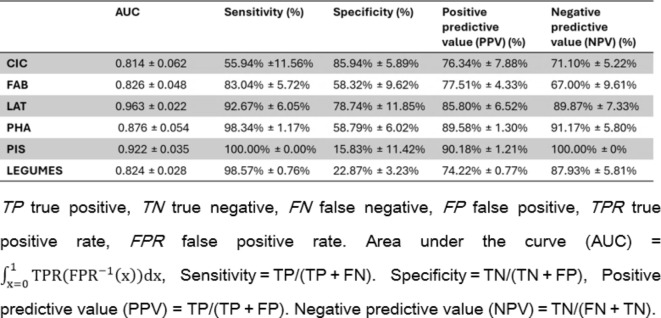
Predictive performance of learning models obtained after cross-validation.

## Discussion

The need to develop novel, non-invasive, easy-to-use, and economic methods for seed quality assessment was the driving force of this work, which proposes the use of UPE and DL as novel tools to evaluate seed quality. To maximize the number of samples with a wider range of germination percentages, this method was tested in a system composed of seeds stored for more than ten years at different conditions (Ambient and Cold) and characterized from the point of view of germination behaviour and photon emission. Following classification in quality classes (optimal, non-optimal), the generated data were used to build prediction models to test the relation between photon emission and seed quality. To our knowledge, currently there is no study that evaluated the UPE and DL phenomena taking into consideration different species and different accessions of the same species. The LIANA© prototype is easy-to-use, fully automated, allowing rapid measurements for diverse purposes covering different surface measurements, while it was not specifically design for seeds. Although the prototype can be further optimized for more accurate analyses on seeds, the costs of its use relate mainly to covering electricity and licensing of the software.

When considering seed germination, several studies performed in legumes^34^, as well as other species^35^, has evidenced differences in G% between storage conditions. In the scenario reflected from the data obtained from pea, beans and lathyrus, the distinction between ambient and cold is less pronounced. The data collected from *P. sativum* can be given as an example from this group. In this case, is appears that seeds stored at both ambient and cold conditions are able to maintain seed germinability in several accessions. A recent study reported similar results in ten varieties of soybeans stored at cold and room temperatures^34^. Other studies showed that pea seeds stored under ambient conditions retained their viability for more than twenty years^31^. In addition, Giannella et al.^36^ reported different germination performance when analysing eight accessions from which one proved to maintain prolonged seed longevity also at room temperature. This accession was characterized by low levels of ROS and increased antioxidant activity and genome stability. Different germination performances between varieties may be explained by other aspects, such as genetic variability that influences plant hormone signalling and other processes related to seed germination^37^.

When considering the results of the machine learning models, these exhibit variable results which can be grouped in two scenarios. The models developed from the datasets CIC and FAB exhibit a moderate efficiency in classifying seed samples appropriately, while the processes obtained from the remaining datasets show high performances. Several factors can affect the quality of prediction and explain the differences in accuracy between the different types of models. For instance, the size of dataset is a crucial issue for machine learning: an optimal training process require an appropriate number of examples representing a wide variety of conditions^38^. The choice of the classifier substantially affects the efficiency of the model. In this study, the predictive models are based on a Random Forest classifier, an ensemble approach widely used for classification tasks that allow the optimization of accuracy and prevent from overfitting of the models^39^. However, an efficient data cleaning phase is important for optimizing the efficiency of the predictive models since the presence of outliers can interfere with the classification^40^. A cost-sensitive classification with the MetaCost operator of RapidMiner has been demonstrated to enhance sensitivity, improving the efficiency of prediction. Thus, this approach may be employed with other models to enhance the balancing of error rates between optimal and non-optimal classes. Apart from the technical aspects related to machine learning, the obtained results support the use of UPE and DL phenomena to estimate seed quality. This is in agreement with other publications indicating that DL and UPE measurements can be used to assess seed viability^9,27^. In addition, the results obtained from the single datasets may suggest the hypothesis of a species-dependent photon emission.

To conclude, seed quality evaluation is a complex aspect since different features (genetic, physiological, and physical factors) are involved in its determination^13^. UPE and DL have been previously correlated to oxidative stress^21^, water content^29^, and seeds vigour^27^, therefore this can be envisioned an accurate method to assess seed quality. The data collected in this work suggests a complex scenario, in which intrinsic seed characteristics of different species may play an important role in the link between seed quality and photon emission. Despite its potential, UPE and DL phenomena require further in-depth characterization to understand their biological relevance in the seed context. The use of machine learning allows to bypass some of the drawbacks related to the lack information about UPE and DL, enabling more accurate prediction of a specific outcome, while contributing to a better understanding of these phenomena.

### Methods

#### Seed materials and storage

Seeds of five legume species, namely bean (*Phaseolus vulgaris* L.), faba bean (*Vicia faba* L.), pea (*Pisum sativum* L.), grass pea (*Lathyrus sativus* L.), and chickpea (*Cicer arietinum* L.), were originated from the genebank collection of the Leibniz-Institute of Plant Genetics and Crop Plant Research (IPK, Gatersleben, Germany) where the material was regenerated under field conditions. Harvest of the seeds was made by hand. After threshing and cleaning the seeds were placed in a drying chamber at a temperature of 22 ± 2 °C and a relative humidity of 11 ± 3% for four weeks. Afterwards the material was divided and transferred either to the cold chamber of the genebank (Cold, sealed glass chars, silica gel on top of the seeds, -18 °C ± 2 °C) or to an ambient storage room (Amb, paper bags, 20 °C ± 2 °C, 50% ± 3% RH).

For each species, 200 seeds per accession were used in the present work. These were divided into distinct seed samples (biological replicates) based on seed size; for *L. sativus* accessions, four replicates of 50 seeds each; for *C. arietinum* and *P. sativum* accessions, five replicates of 40 seeds each; for *P. vulgaris* and *V. faba* accessions, eight replicates of 25 seeds each. Different number of accessions per species were used as follows: 22 accession for *P. vulgaris*, *P. sativum*, and *C. arietinum*, 12 accession for *L. sativus*, and 11 accessions for *V. faba*. The time of storage was selected based on previous seed bank analyses carried out to identify the most suitable conditions where contrastive germination performance could be observed^31^. The accessions used here were collected at different harvest years: 2010 for *L. sativus* and *P. sativum*, 2012 for *P. vulgaris*, and 2013 for *V. faba* and *C. arietinum*.

## Germination parameters

Germination tests were performed following the guidelines provided by ISTA (International Rules for Seed Testing (https://www.seedtest.org/) with some modifications. The conditions for each species/accession were as follows: for *P. vulgaris*, and *C. arietinum* seed were germinated at 25 °C for 8 days; for *P. sativum* seed were germinated at 20 °C for 8 days; for *V. faba* and *L. sativus* seed were germinated at 20 °C for 10 days. For *V. faba*, seeds were maintained at 4 °C for 7 days before starting the germination test. Seeds stored at Amb and Cold conditions were monitored in parallel. Different groups of seeds (*L. sativus*, 4 replicates of 50 seeds/replicate; *P. sativum* and *C. arietinum*, 5 replicates of 40 seeds/replicate; *V. faba* and *P. vulgaris*, 8 replicates of 25 seeds/replicate) were placed in germination trays containing filter paper moistened with distilled water. All containers were kept in a growth chamber at the indicated temperatures under 16 h dark/8 h light. At the end of germination, the following germination indices were calculated: germination percentage (G), mean germination time (MGT), and synchronicity index (Z)^41^. The formulas used for the calculation of these parameters are the following.

1. $$\:\text{G}\text{\%}=\left(\frac{number\:of\:germinated\:seeds)}{total\:number\:of\:seeds}\right)\times\:100\:$$
2. $$\:\:MGT=\frac{\sum\:_{i=1}^{k}ni\:x\:ti}{\sum\:_{i=1}^{k}ni}$$
3. $$\:\:Z=\frac{\sum\:Cni,2}{\sum\:ni\:x\:\sum\:\left(ni-\frac{1}{2}\right)}$$

In MGT (2) and Z (3) formulas, *ni* is the number of seeds germinated in the time *i* (not the accumulated number, but the number correspondent to the *ith* observation), *ti* corresponds to the time from the start of the experiment to the *ith* observation (day), *k* is the last time of germination, and *Cni*,*2* = *ni* (*ni*-1)/2. Germination data were analysed with Student *t*-test using the Microsoft Excel package using as threshold the *p*-value ≤ 0.05 (‘*’).

Seedling growth was monitored on the final day of the germination test by using ImageJ (https://imagej.nih.gov/ij/) software. For each accession and replicate, 20% of the seedlings were photographed and used to determine the seedling length in terms of roots and/or aerial parts.

### Statistical analyses

Germination and physical traits data were analysed with Student *t*-test using the Microsoft Excel package using as threshold the *p*-value ≤ 0.05 (‘*’). For correlation analyses, Pearson’s correlation coefficient and the relative p-values were determined by using MetaboAnalyst 6.0 (https://www.metaboanalyst.ca/)^42^. The same software was also used for principal component analysis (PCA) performed by using all the germination parameters. The obtained “score plot” graphics show how the different sample groups are clustered according to the results obtained in the performed analyses.

### UPE and DL detection

**Fig. 7:**
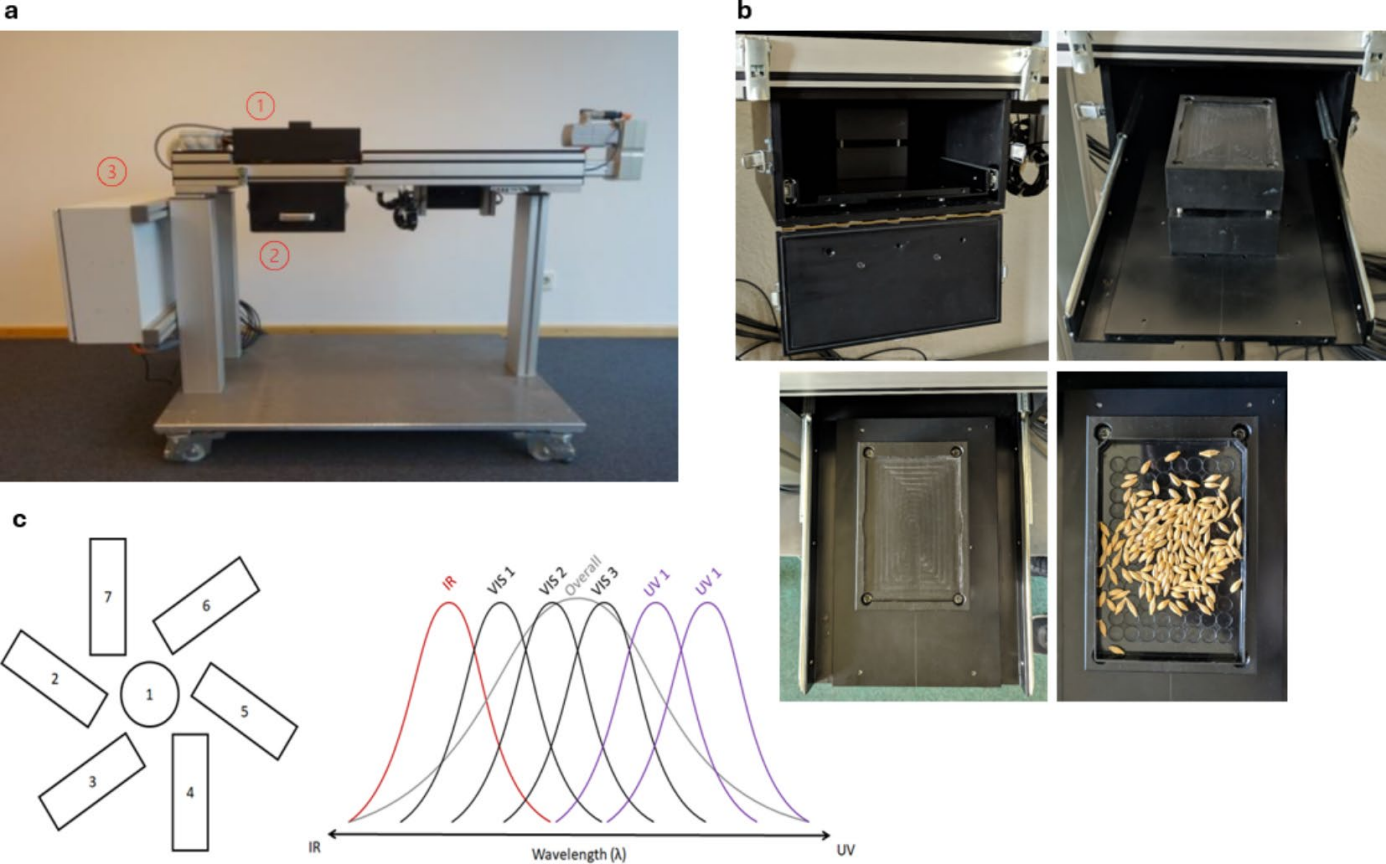
Representative images showing the structure of the LIANA© device and its use for light analysis on seeds. (**a**) The main structure of the LIANA© device illustrating (1) the excitation source, which includes the PMTs (photomultiplier tubes), (2) the sample chamber, and (3) the electrical block. (**b**) The seed chamber. It can be opened by releasing the clips on either sides (top left). The front door can be drawn down to access the internal drawer with the sample holding block (top right). The drawer must be pulled out completely while inserting the seeds into the tray for the analysis (bottom). (**c**) The excitation source (UV) and the PMTs. These include seven photomultiplier tubes along with filters to form seven sensors which can detect the emission at different wavelengths.

A light analyzer (LIANA©, SuperLab, Käthe-Kruse-Str. 11, 26160 Bad Zwischenahn, Germany, https://www.super-lab.de//liana.html) has been used to collect UPE and DL data of seed samples. This prototype is covered by the SuperLab IP copyright patent number EP 2,613,139 A1. The LIANA© prototype (Fig. 7) contains seven photomultiplier tubes along with filters to form seven sensors that detect light emission at different wavelengths. The characteristics of the photomultiplier tubes (PMTs, Hamamatsu Photonics), including model type, spectrum and filter bandpass wavelength are presented in Supplementary Table S6. For the DL excitation, 6 LED with the spectral range 380–420 nm were used. The measurements were conducted with the following parameters: time, 10 (10 s measurement time); frequency, 100 (measure every 0.1 s); size, 100; distance, 10; surface, 10; illumination time, 1 (1-second illumination); darkcount time, 5 (5 s darkcount data before and after each measurement); dark-count frequency, 100 (measure every 0.1 s). For each sample, measures were taken five times (technical replicates), from which eventual outliers have been eliminated. At least three technical replicates have been retained for each seed sample. For a more realistic photon count estimation, a darkcount measurement is automatically performed before and after each measurement, averaged and subtracted from the values of the PMTs (the photon counted during the measurement time). Then a “real amount of photons” is calculated by multiplying this new value by correction factors, based on the position of the PMTs, the filter used, and the overlapping of wavelengths with other PMTs. In addition to the corrected photon counts, a large number of features reflecting the UPE and DL phenomena are acquired and included in the datasets.

### Generation of prediction models

**Fig. 8:**
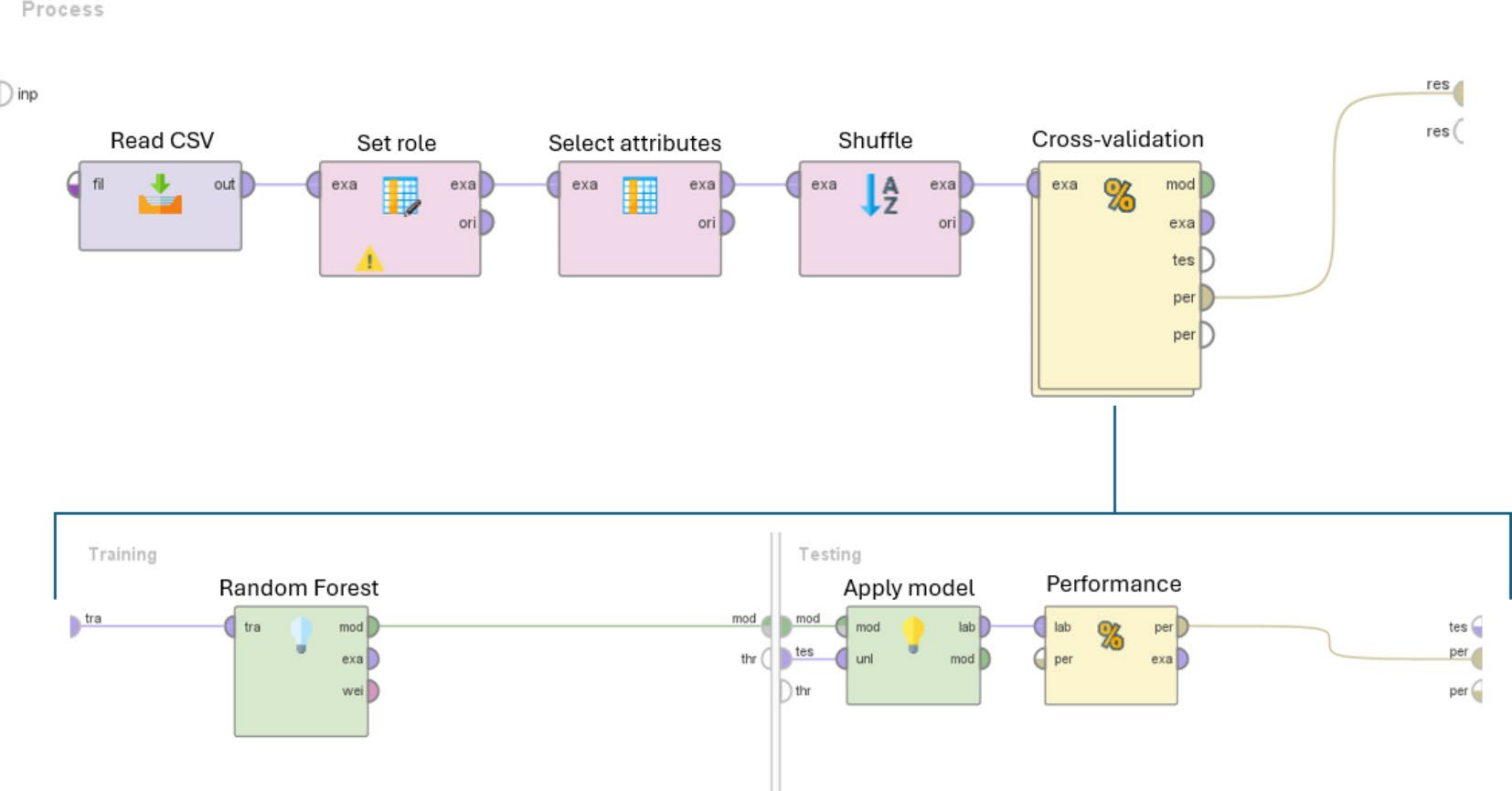
Illustration of the RapidMiner prediction model. The operators displayed in the model are connected and execute distinct actions. The Read CSV operator allows the uploading of the file. To facilitate the manual integration of germination percentages into the CSV file, the records were sorted. The Set role operator was used for data labelling. Attributes highly correlated to the label were excluded from the learning model with the operator Select attributes. The operator Shuffle was employed to randomize the records within the datasets.

Prediction models for the classification of seed samples were assigned and improved using the RapidMiner software^43,44^. Seeds were classifieds based on G% values into optimal (100 − 80%) and non-optimal (below 80%) quality. The prediction process is described in Fig. 8. The several operators used in the learning process are connected in a specific order and perform different operations. A stratified 10-fold cross-validation operation approach was used to validate the model. The classifier Random Forest was selected for outcome prediction to maximize accuracy. Accuracy %, area under the curve (AUC), sensitivity %, specificity %, positive predicted value (PPV) %, and negative predicted value (NPV) % values were obtained at the end of the validation.

## Electronic supplementary material

Below is the link to the electronic supplementary material.

Supplementary Material 1

Supplementary Material 2

## Data Availability

The datasets used and/or analysed during the current study are available from the corresponding author on reasonable request.

## References

[1] Huang, M., Wang, Q. G., Zhu, Q. B., Qin, J. W. & Huang, G. Review of seed quality and safety tests using optical sensing technologies. *SST ***43** (3), 337–366 (2015).

[2] www.thebusinessresearchcompany.com. (n.d.). *Seeds Market Size, Share, Growth, Trend Analysis, Forecast 2033*. https://www.thebusinessresearchcompany.com/report/seeds-global-market-report (2024).

[3] McDonald, M. B. Assessment of seed Quality1. *HortScience ***15** (6), 784–788 (1980).

[4] McDonald, M. B. Seed quality assessment. *Seed Sci. Res. ***8** (2), 265–276 (1998).

[5] Elizalde, V. et al. Viability and germination of *Hechtia perotensis* (*Bromeliaceae*) seed. *Rev. Biol. Trop. ***65** (1), 153–165 (2017). 29466635

[6] Ureña, R., Rodríguez, F. & Berenguel, M. A machine vision system for seeds quality evaluation using fuzzy logic. *Comput. Electron. Agric. ***32** (1), 1–20 (2001).

[7] Lin, P. et al. Rapidly and exactly determining postharvest dry soybean seed quality based on machine vision technology. *Sci. Rep. ***9** (1), 17143 (2019). 31748535 10.1038/s41598-019-53796-w PMC6868226

[8] Zhu, L. et al. Advances of NIR spectroscopy technology applied in seed quality detection. *Spectrosc. Spect. Anal. ***35** (2), 346–349 (2015). 25970890

[9] Li, W., Tan, F., Cui, J. & Ma, B. Fast identification of soybean varieties using Raman spectroscopy. *Vib. Spectrosc. ***123**, 103447 (2022).

[10] Feng, L. et al. Hyperspectral imaging for seed quality and safety inspection: a review. *Plant. Methods ***91** (1), 15 (2019). 10.1186/s13007-019-0476-y PMC6686453 31406499

[11] ElMasry, G. et al. Emerging thermal imaging techniques for seed quality evaluation: principles and applications. *Food Res. Int. ***131**, 109025 (2020). 32247450 10.1016/j.foodres.2020.109025

[12] Musaev, F., Priyatkin, N., Potrakhov, N., Beletskiy, S. & Chesnokov, Y. Assessment of *Brassicaceae* seeds Quality by X-ray analysis. *Hortic ***8** (1), 29 (2022).

[13] Rahman, A. & Cho, B. K. Assessment of seed quality using non-destructive measurement techniques: a review. *Seed Sci. Res. ***26** (4), 285–305 (2016).

[14] Du, J. et al. The application and trend of ultra-weak photon emission in biology and medicine. *Fchem ***11**, 1140128 (2023). 10.3389/fchem.2023.1140128 PMC9981976 36874066

[15] Cifra, M. & Pospíšil, P. Ultra-weak photon emission from biological samples: definition, mechanisms, properties, detection and applications. *J. Photochem. Photobiol B ***139**, 2–10 (2014). 24726298 10.1016/j.jphotobiol.2014.02.009

[16] Cilento, G. & Adam, W. From free radicals to electronically excited species. *Free Radic Biol. Med. ***19** (1), 103–114 (1995). 7635351 10.1016/0891-5849(95)00002-f

[17] Wang, C., Bókkon, I., Dai, J. & Antal, I. Spontaneous and visible light-induced ultraweak photon emission from rat eyes. *Brain Res. ***1369**, 1–9 (2011). 21034725 10.1016/j.brainres.2010.10.077

[18] Popp, F. A. & Yan, Y. Delayed luminescence of biological systems in terms of coherent states. *Phys. Lett. ***293** (1–2), 93–97 (2002).

[19] Goltsev, V., Zaharieva, I., Chernev, P. & Strasser, R. J. Delayed fluorescence in photosynthesis. *Photosynth Res. ***101**, 217–232 (2009). 19548111 10.1007/s11120-009-9451-1

[20] Kobayashi, M. Highly sensitive imaging for ultra-weak photon emission from living organisms. *J. Photochem. Photobiol B ***139**, 34–38 (2014). 24360927 10.1016/j.jphotobiol.2013.11.011

[21] Pospíšil, P., Prasad, A. & Rác, M. Role of reactive oxygen species in ultra-weak photon emission in biological systems. *J. Photochem. Photobiol B ***139**, 11–23 (2014). 24674863 10.1016/j.jphotobiol.2014.02.008

[22] Sun, C., Liu, J., Liu, H. & Guo, J. Reactive oxygen species mediate the relationship between mitochondrial function and delayed luminescence during senescence of strawberry (*Fragaria ananassa*) fruits. *Acta Physiol. Plant. ***44**(2) (2022).

[23] Zhang, J. et al. Roles of NOD1/*Rip2* signal pathway in carotid artery remodelling in spontaneous hypertensive rats. *Gen. Physiol. Biophys. ***41** (01), 31–42 (2022). 35253648 10.4149/gpb_2021042

[24] Murphy, M. P. et al. Unraveling the Biological roles of reactive oxygen species. *Cell. Metab. ***13** (4), 361–366 (2011). 21459321 10.1016/j.cmet.2011.03.010 PMC4445605

[25] Griffo, A., Bosco, N., Pagano, A., Balestrazzi, A. & Macovei, A. Noninvasive methods to detect reactive oxygen species as a proxy of seed quality. *Antioxidants ***12** (3), 626 (2023). 36978875 10.3390/antiox12030626 PMC10045522

[26] Stolz, P., Wohlers, J. & Mende, G. Measuring delayed luminescence by FES to evaluate special quality aspects of food samples – an overview. *Open. Agric. ***4** (1), 410–417 (2019).

[27] Grasso, R. et al. Non-destructive evaluation of watermelon seeds germination by using delayed luminescence. *J. Photochem. Photobiol. B ***187**, 126–130 (2018). 30145462 10.1016/j.jphotobiol.2018.08.012

[28] Adeboye, K. & Börner, A. Delayed luminescence of seeds: are shining seeds viable? *SST ***48** (2), 167–177 (2020).

[29] Yan, Y., Popp, F. A. & Rothe, G. M. Correlation between germination capacity and biophoton emission of barley seeds (*Hordeum vulgare* L). *SST ***31** (2), 249–258 (2003).

[30] Costanzo, E. et al. Single seed viability checked by delayed luminescence. *EBJ ***37** (2), 235–238 (2007). 17952430 10.1007/s00249-007-0221-8

[31] Nagel, M. & Börner, A. The longevity of crop seeds stored under ambient conditions. *Seed Sci. Res. ***20** (1), 1–12 (2010).

[32] Kim, J., Choi, K., Kim, G. & Suh, Y. Classification cost: an empirical comparison among traditional classifier, cost-sensitive classifier, and MetaCost. *Expert Syst. Appl. ***39** (4), 4013–4019 (2012).

[33] Wang, Y. C. & Cheng, C. H. A multiple combined method for rebalancing medical data with class imbalances. *Comput. Biol. Med. ***134**, 104527 (2021). 34091384 10.1016/j.compbiomed.2021.104527

[34] Koskosidis, A., Khah, E. M., Pavli, O. I. & Vlachostergios, D. N. Effect of storage conditions on seed quality of soybean (*Glycine max* L.) *g*ermplasm. *AIMS Agric. Food ***7** (2), 387–402 (2022).

[35] Agacka, M. et al. Viability of *Nicotiana* spp. seeds stored under ambient temperature. *SST ***41** (3), 474–478 (2013).

[36] Gianella, M. et al. Physiological and molecular aspects of seed longevity: exploring intra-species variation in eight *Pisum sativum* L. accessions. *Physiol. Plant. ***174**(3), e13698. (2022). 10.1111/ppl.13698 PMC9321030 35526223

[37] Miransari, M. & Smith, D. L. Plant hormones and seed germination. *EEB ***99**, 110–121 (2014).

[38] Barbedo, J. G. A. Impact of dataset size and variety on the effectiveness of deep learning and transfer learning for plant disease classification. *Comput. Electron. Agr. ***153**, 46–53 (2018).

[39] Rodriguez-Galiano, V. F., Ghimire, B., Rogan, J., Chica-Olmo, M. & Rigol-Sanchez, J. P. An assessment of the effectiveness of a random forest classifier for land-cover classification. *P&RS ***67**, 93–104 (2012).

[40] Fernández, Á., Bella, J. & Dorronsoro, J. R. Supervised outlier detection for classification and regression. *Neurocomputing ***486**, 77–92 (2022).

[41] Ranal, M. A. & de Santana, D. G. How and why to measure the germination process? *Rev. Bras. Bot. ***29** (1), 1–11 (2006).

[42] Pang, Z. et al. MetaboAnalyst 6.0: towards a unified platform for metabolomics data processing, analysis and interpretation. *Nucleic Acids Res.* gkae253 (2024). 10.1093/nar/gkae253 PMC11223798 38587201

[43] Kotu, V. & Deshpande, B. *Predictive Analytics and Data Mining* (Elsevier Science & Technology, 2014).

[44] Hofmann, M., Klinkenberg, R. & RapidMiner *Data Mining use Cases and Business Analytics Applications* (CRC, 2016).

