# Supplementary material for: Non-invasive methods to assess seed quality based on ultra-weak photon emission and delayed luminescence
Source: Sci Rep. 2024 Nov 5;14:26838. doi: 10.1038/s41598-024-74207-9 (PMC11538308; doi:10.1038/s41598-024-74207-9)
Supplement: Supplementary file 1 — Supplementary Material 1 [file 41598_2024_74207_MOESM1_ESM.docx]

**Non-invasive methods to assess seed quality based on ultra-weak photon emission and delayed luminescence**

Adriano Griffo^1^, Stefanie Sehmisch^2^, Frédéric Laager^3^, Andrea Pagano^1^, Alma Balestrazzi^1^, Anca Macovei^1^, Andreas Börner^2^

^1^ Department of Biology and Biotechnology ‘L. Spallanzani’, University of Pavia, Pavia, PV 27100, Italy

^2^ Leibniz Institute of Plant Genetics and Crop Plant Research, Seeland, Saxony-Anhalt 06466, Germany

^3^ SUPER Lab, Bad Zwischenahn, Bad Zwischenahn 26160, Germany

**Corresponding authors:**

Anca Macovei; Address: Via Ferrata 9, 27100 Pavia, Italy; E-mail: [anca.macovei@unipv.it](mailto:anca.macovei@unipv.it); Phone: 0382 985583; Fax: +39-0382985583

Andreas Börner: Address: Corrensstr. 3, 06466 Gatersleben, Germany; E-mail: [boerner@ipk-gatersleben.de](mailto:boerner@ipk-gatersleben.de); Phone: +49 394 825 229; Fax: +49 394 825 155

**Supplementary Information**

**Table S1.** List of accessions used in the present work for *P. sativum*. For each accession, year of harvest, time of storage, genus, species, spauthor, and origin are indicated.

| **Accession** | **Year of harvest** | **Time of storage (years)** | **Genus** | **Species** | **Spauthor** | **Origin** |
| --- | --- | --- | --- | --- | --- | --- |
| PIS 14 | 2010 | 13 | *Pisum* | *sativum* | L. | Germany |
| PIS 17 | 2010 | 13 | *Pisum* | *sativum* | L. | Germany |
| PIS 40 | 2010 | 13 | *Pisum* | *sativum* | L. | Germany |
| PIS 41 | 2010 | 13 | *Pisum* | *sativum* | L. | Germany |
| PIS 108 | 2010 | 13 | *Pisum* | *sativum* | L. | Greece |
| PIS 213 | 2010 | 13 | *Pisum* | *sativum* | L. | Germany |
| PIS 493 | 2010 | 13 | *Pisum* | *sativum* | L. | Afghanistan |
| PIS 1135 | 2010 | 13 | *Pisum* | *sativum* | L. | Union of Soviet Socialist Republics |
| PIS 5049 | 2010 | 13 | *Pisum* | *sativum* | L. | Czech Republic |
| PIS 7226 | 2010 | 13 | *Pisum* | *sativum* | L. | Union of Soviet Socialist Republics |
| PIS 7238 | 2010 | 13 | *Pisum* | *sativum* | L. | Union of Soviet Socialist Republics |
| PIS 7243 | 2010 | 13 | *Pisum* | *sativum* | L. | Union of Soviet Socialist Republics |
| PIS 7246 | 2010 | 13 | *Pisum* | *sativum* | L. | Union of Soviet Socialist Republics |
| PIS 7253 | 2010 | 13 | *Pisum* | *sativum* | L. | Poland |
| PIS 7261 | 2010 | 13 | *Pisum* | *sativum* | L. | Union of Soviet Socialist Republics |
| PIS 7264 | 2010 | 13 | *Pisum* | *sativum* | L. | Union of Soviet Socialist Republics |
| PIS 7271 | 2010 | 13 | *Pisum* | *sativum* | L. | Union of Soviet Socialist Republics |
| PIS 7365 | 2010 | 13 | *Pisum* | *sativum* | L. | Georgia |
| PIS 7560 | 2010 | 13 | *Pisum* | *sativum* | L. | Unknown |
| PIS 7569 | 2010 | 13 | *Pisum* | *sativum* | L. | Tunisia |
| PIS 7760 | 2010 | 13 | *Pisum* | *sativum* | L. | Germany |

**Table S2.** List of accessions used in the present work for *C. arietinum*. For each accession, year of harvest, time of storage, genus, species, spauthor, and origin are indicated.

| **Accession** | **Year of harvest** | **Time of storage (years)** | **Genus** | **Species** | **Spauthor** | **Origin** |
| --- | --- | --- | --- | --- | --- | --- |
| CIC 5 | 2013 | 10 | *Cicer* | *arietinum* | L. | Turkey |
| CIC 18 | 2013 | 10 | *Cicer* | *arietinum* | L. | Greece |
| CIC 28 | 2013 | 10 | *Cicer* | *arietinum* | L. | Greece |
| CIC 31 | 2013 | 10 | *Cicer* | *arietinum* | L. | Unknown |
| CIC 49 | 2013 | 10 | *Cicer* | *arietinum* | L. | Union of Soviet Socialist Republics |
| CIC 50 | 2013 | 10 | *Cicer* | *arietinum* | L. | Union of Soviet Socialist Republics |
| CIC 53 | 2013 | 10 | *Cicer* | *arietinum* | L. | Union of Soviet Socialist Republics |
| CIC 58 | 2013 | 10 | *Cicer* | *arietinum* | L. | Unknown |
| CIC 90 | 2013 | 10 | *Cicer* | *arietinum* | L. | Italy |
| CIC 92 | 2013 | 10 | *Cicer* | *arietinum* | L. | Italy |
| CIC 94 | 2013 | 10 | *Cicer* | *arietinum* | L. | Italy |
| CIC 180 | 2013 | 10 | *Cicer* | *arietinum* | L. | Tajikistan |
| CIC 204 | 2013 | 10 | *Cicer* | *arietinum* | L. | Tajikistan |
| CIC 205 | 2013 | 10 | *Cicer* | *arietinum* | L. | Tunisia |
| CIC 209 | 2013 | 10 | *Cicer* | *arietinum* | L. | Albania |
| CIC 210 | 2013 | 10 | *Cicer* | *arietinum* | L. | Albania |
| CIC 221 | 2013 | 10 | *Cicer* | *arietinum* | L. | Italy |
| CIC 648 | 2013 | 10 | *Cicer* | *arietinum* | L. | India |
| CIC 694 | 2013 | 10 | *Cicer* | *arietinum* | L. | Tunisia |
| CIC 701 | 2013 | 10 | *Cicer* | *arietinum* | L. | Iran |
| CIC 702 | 2013 | 10 | *Cicer* | *arietinum* | L. | Iran |
| CIC 737 | 2013 | 10 | *Cicer* | *arietinum* | L. | Italy |

**Table S3.** List of accessions used in the present work for *V. faba*. For each accession, year of harvest, time of storage, genus, species, spauthor, and origin are indicated.

| **Accession** | **Year of harvest** | **Time of storage (years)** | **Genus** | **Species** | **Spauthor** | **Origin** |
| --- | --- | --- | --- | --- | --- | --- |
| FAB 82 | 2013 | 10 | *Vicia* | *faba* | L. | Germany |
| FAB 129 | 2013 | 10 | *Vicia* | *faba* | L. | Czech Republic |
| FAB 571 | 2013 | 10 | *Vicia* | *faba* | L. | Spain |
| FAB 6146 | 2013 | 10 | *Vicia* | *faba* | L. | Japan |
| FAB 6956 | 2013 | 10 | *Vicia* | *faba* | L. | Spain |
| FAB 6975 | 2013 | 10 | *Vicia* | *faba* | L. | Italy |
| FAB 6997 | 2013 | 10 | *Vicia* | *faba* | L. | Italy |
| FAB 6999 | 2013 | 10 | *Vicia* | *faba* | L. | Italy |
| FAB 7200 | 2013 | 10 | *Vicia* | *faba* | L. | Italy |
| FAB 7458 | 2013 | 10 | *Vicia* | *faba* | L. | Spain |
| FAB 7479 | 2013 | 10 | *Vicia* | *faba* | L. | Croatia |

**Table S4.** List of accession used in the present work for *L. sativus*. For each accession, year of harvest, time of storage, genus, species, spauthor, and origin are indicated.

| **Accession** | **Year of harvest** | **Time of storage (years)** | **Genus** | **Species** | **Spauthor** | **Origin** |
| --- | --- | --- | --- | --- | --- | --- |
| LAT 227 | 2010 | 13 | *Lathyrus* | *cicera* | L. | Greece |
| LAT 237 | 2010 | 13 | *Lathyrus* | *cicera* | L. | Greece |
| LAT 412 | 2010 | 13 | *Lathyrus* | *sativus* | L. | Greece |
| LAT 416 | 2010 | 13 | *Lathyrus* | *sativus* | L. | Greece |
| LAT 434 | 2010 | 13 | *Lathyrus* | *sativus* | L. | Ukraine |
| LAT 438 | 2010 | 13 | *Lathyrus* | *sativus* | L. | Unknown |
| LAT 443 | 2010 | 13 | *Lathyrus* | *sativus* | L. | Unknown |
| LAT 455 | 2010 | 13 | *Lathyrus* | *sativus* | L. | Hungary |
| LAT 456 | 2010 | 13 | *Lathyrus* | *sativus* | L. | Spain |
| LAT 457 | 2010 | 13 | *Lathyrus* | *sativus* | L. | Iran |
| LAT 458 | 2010 | 13 | *Lathyrus* | *sativus* | L. | Iran |
| LAT 468 | 2010 | 13 | *Lathyrus* | *sativus* | L. | Iran |

**Table S5.** List of accessions used in the present work for *P. vulgaris*. For each accession, year of harvest, time of storage, genus, species, spauthor, and origin are indicated.

| **Accession** | **Year of harvest** | **Time of storage (years)** | **Genus** | **Species** | **Spauthor** | **Origin** |
| --- | --- | --- | --- | --- | --- | --- |
| PHA 99 | 2012 | 11 | *Phaseolus* | *vulgaris* | L. | Greece |
| PHA 100 | 2012 | 11 | *Phaseolus* | *vulgaris* | L. | Greece |
| PHA 161 | 2012 | 11 | *Phaseolus* | *vulgaris* | L. | Poland |
| PHA 167 | 2012 | 11 | *Phaseolus* | *vulgaris* | L. | Greece |
| PHA 182 | 2012 | 11 | *Phaseolus* | *vulgaris* | L. | Greece |
| PHA 254 | 2012 | 11 | *Phaseolus* | *vulgaris* | L. | Unknown |
| PHA 309 | 2012 | 11 | *Phaseolus* | *vulgaris* | L. | Greece |
| PHA 386 | 2012 | 11 | *Phaseolus* | *vulgaris* | L. | Unknown |
| PHA 390 | 2012 | 11 | *Phaseolus* | *vulgaris* | L. | Unknown |
| PHA 416 | 2012 | 11 | *Phaseolus* | *vulgaris* | L. | Unknown |
| PHA 419 | 2012 | 11 | *Phaseolus* | *vulgaris* | L. | Switzerland |
| PHA 507 | 2012 | 11 | *Phaseolus* | *vulgaris* | L. | Bulgaria |
| PHA 673 | 2012 | 11 | *Phaseolus* | *vulgaris* | L. | Unknown |
| PHA 869 | 2012 | 11 | *Phaseolus* | *vulgaris* | L. | Greece |
| PHA 942 | 2012 | 11 | *Phaseolus* | *vulgaris* | L. | Italy |
| PHA 1448 | 2012 | 11 | *Phaseolus* | *vulgaris* | L. | Slovakia |
| PHA 1450 | 2012 | 11 | *Phaseolus* | *vulgaris* | L. | Sklovakia |
| PHA 6017 | 2012 | 11 | *Phaseolus* | *vulgaris* | L. | France |
| PHA 6018 | 2012 | 11 | *Phaseolus* | *vulgaris* | L. | Netherlands |
| PHA 6019 | 2012 | 11 | *Phaseolus* | *vulgaris* | L. | Australia |
| PHA 6020 | 2012 | 11 | *Phaseolus* | *vulgaris* | L. | Netherlands |
| PHA 6021 | 2012 | 11 | *Phaseolus* | *vulgaris* | L. | United States of America |
| PHA 6022 | 2012 | 11 | *Phaseolus* | *vulgaris* | L. | United States of America |

**Supplementary Table S6**. Features of the photomultiplier tubes (PMTs) of the LIANA© device, including model type, spectrum and filter bandpass wavelength.

| No. | 1 | 2 | 3 | 4 | 5 | 6 | 7 |
| --- | --- | --- | --- | --- | --- | --- | --- |
| PMT Model | **R1924P** | R3788 | R3788 | **R7154** | R3788 | **R3896** | **R3896** |
| PMT spectrum nm | **300-650** | 185-750 | 185-750 | **160-320** | 185-750 | **185-900** | **185-900** |
| Filter bandpass  Wavelength nm | No Filter | **280-310** | **315-400** | No Filter | **430-510** | **530-610** | 610-690 |

**
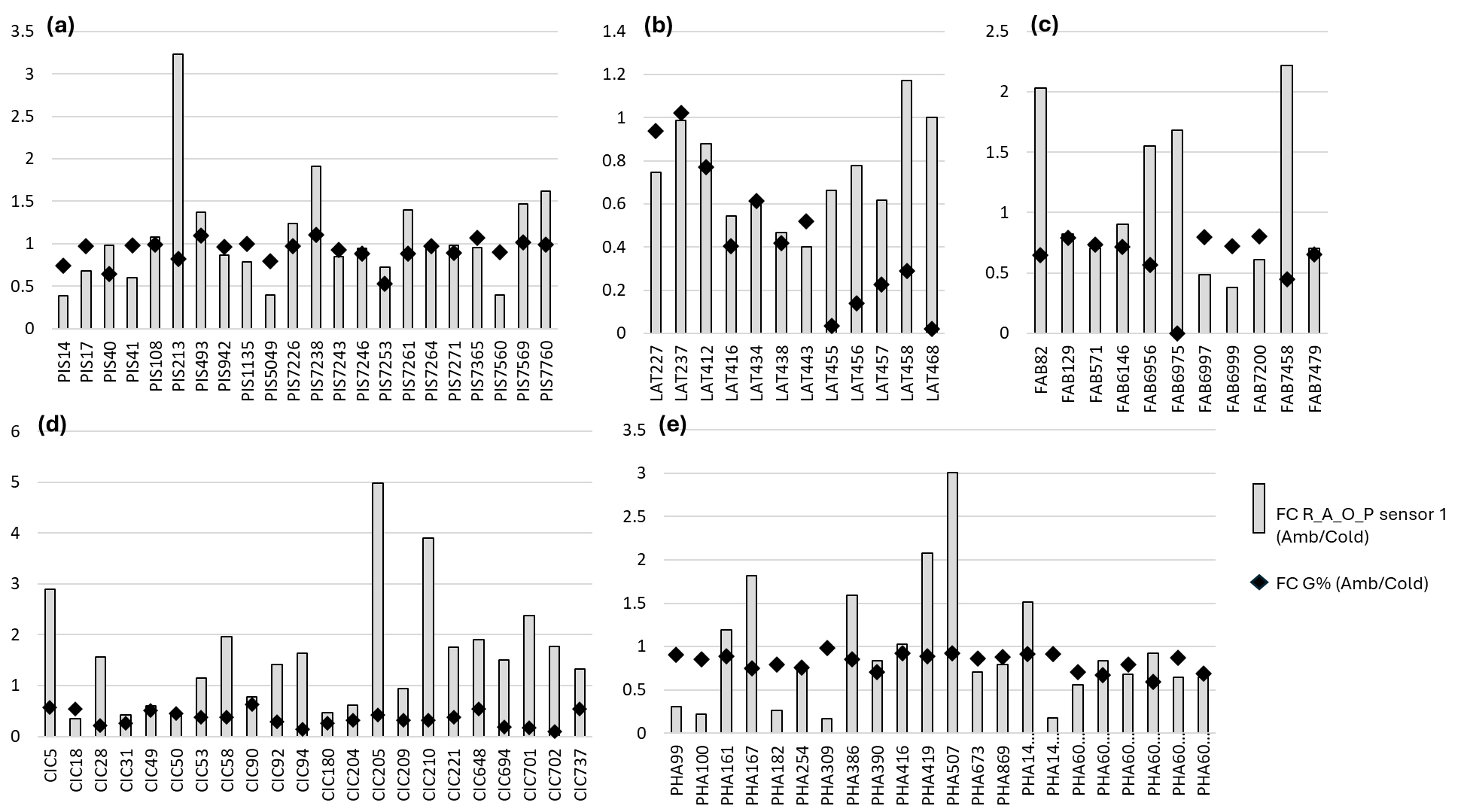
**

**Supplementary Figure 1**. Representation of the total amount of photons counted through sensor 1 of the LIANA© prototype during measurement time (real amount of photon, RAOP sensor 1, grey histograms) in relation to the percentage of germination (G%, black rhomboids) for each accession of *P. sativum* **(a)**, *L. sativus* **(b)**, *V. faba* **(c)**, *C. arietinum* **(d)**, and *P. vulgaris* **(e)**. The values are presented in the form of fold-change (FC) (Amb/Cold).
